# Barriers to access to hepatitis C treatment with direct-acting antivirals in people who inject drugs in the community setting

**DOI:** 10.1186/s12954-024-01009-7

**Published:** 2024-04-27

**Authors:** Elena Yela, Neus Solé, Lidia Puig, Darío López Gallegos, Rafael Clua-García

**Affiliations:** 1 Sant Esteve Sesrovires Prison Health Care Team (Brians 1 Prison Centre), South Metropolitan Health Care Department, Catalan Health Institute, Sant Esteve Sesrovires, Barcelona, Spain; 2 Fundació Institut Universitari per a la recerca a l’Atenció Primària de Salut Jordi Gol i Gurina (IDIAPJGol), Barcelona, Spain; 3 South Metropolitan Health Care Department, Catalan Health Institute, Barcelona, Spain; 4 Manresa Faculty of Health Sciences, University of Vic - Central University of Catalonia, Manresa, Spain

**Keywords:** Hepatitis C, People who inject drugs, Direct-acting antivirals, Harm reduction, Qualitative, Spain

## Abstract

Barriers to access to hepatitis C treatment with direct-acting antivirals in people who inject drugs in the community setting. Qualitative study with prison population. Hepatitis C (HCV) treatments with direct-acting antiviral therapy (DAA) are an easy and effective option among people who inject drugs (PWID). However, difficulties in accessing and monitoring treatment in community services and dropouts on release from prison are detected among PWID. For this reason, the aim of the study is to know the access barriers in the diagnosis and treatment of HCV in community health services. An exploratory qualitative study was carried out through semi-structured interviews with 33 PWID recruited in a pre-trial detention prison in Barcelona. The information obtained was analysed using grounded theory. Among PWID sub-population entering prison, personal barriers are related to intense drug use, lack of interest and ignorance of HCV infection and treatment, as well as being in a situation of social exclusion. In relation to health providers, they reported receiving little information, the existence of language barriers in migrants, not receiving screening and treatment proposals, and having poor interactions with some professionals. Systemic barriers were expressed related to the health system circuit being complicated, perceiving little comprehensive care and lack of community support. It is recommended to intensify prevention and treatment campaigns, promoting drug substitution programmes than current ones, improve health education, make the diagnosis and treatment process more flexible, and promote social policies and holistic care for greater coverage of the needs of PWID.

## Introduction

Hepatitis C elimination has been a public health challenge for the World Health Organization (WHO) since 2016, whose goals are to reduce incident cases by 90% and mortality by 65% by 2030 [1–3]. As strategic measures to achieve these objectives by intensifying diagnosis and facilitating access to treatment with direct-acting antivirals (DAAs), in groups that present risk factors and difficulties in accessing health services, it is proposed to apply a set of harm reduction measures, as well as tailoring programmes to different population groups, linking and integrating HCV-specific services across different health services and strengthening community services as they provide an opportunity to reach marginalised groups and increase acceptability and the use of services [3].

Second-generation DAAs have been one of the main facilitators for HCV treatment given their efficacy, convenience and tolerance [4]. However, people who inject drugs (PWID) present difficulties in accessing HCV diagnosis and treatment in regular circuits due to their situation of exclusion and social precariousness [5, 6]. It has been shown that PWID who create adherence to treatment have cure rates of more than 90% [7, 8] which is why it is necessary to persevere in interventions to facilitate access to diagnosis and DAAs in this group.

Catalonia is a region of Spain that records a high incidence of HCV among PWID and an estimated prevalence of between 65 and 80% of the infected population [9–11]. Following the WHO guidelines, the Department of Health established an HCV prevention and control plan, placing special interest in this group [12], which has involved changes to increase the effectiveness of the detection and treatment system through universal screening, “single-step” diagnosis and bringing specialists closer to the drug-dependence health services [10, 13]. The health coverage of this group is also guaranteed through an exceptional circuit of the General Sub-directorate for Drug Dependence for cases in which people do not have a health card [14]. Specifically, detection has been strengthened from harm reduction services in Catalonia, included within care for drug dependencies. These services offer needle exchange programmes (NEP), drug consumption rooms (DCR) and health and social care, to which PWID who present social and health difficulties attend to access diagnosis and treatment in other health care services or through ordinary circuits [15, 16]. Recent studies carried out in Catalonia highlight that around 35% of PWID present hidden infection, with a higher percentage among migrants [11, 13].

In parallel, prisons in Catalonia, as elsewhere in the rest of Spain, have proven to be ideal settings for detecting HCV infection through early screening and the availability of DAA treatments [8, 17, 18]. A high percentage of PWID from the community circulate in the prison environment and various studies have detected that around 70% of this group has problems with the law, having had several admissions to prison centres [9, 19]. In fact, a significant number of people with HCV were detected in pre-trial detention admissions in Catalan prisons, of whom 88% had a history of PWID [20], detecting 2.7% of cases with active RNA + infection coming from the community [21]. Hidden infection was detected among 23.7% of these and, the same as in the community [11, 13], mainly in the immigrant population [20].

Several studies have described a positive response to DAA intervention, showing high adherence and high cure rates, greater than 90% sustained virological response (SVR) [8, 18, 20]. However, it was detected in Catalan prisons that three out of seven HCV treatment abandonments are due to release [8]. Therefore, to ensure continuity of care and treatment of chronic health problems in prison, including blood-borne infections (HIV, HCV, etc.), the liaison nurse figure has been incorporated into the prison health system as a mechanism for effective coordination between in-prison and extra-prison health services [20, 22].

In Catalonia, despite the significant deployment of strategies to improve access to diagnosing and treating HCV with DAA among PWID, abandonment and lack of adherence to follow-up in health care services and drug dependence health services continue to be detected in the community or when leaving prison. However, although the characteristics/profile of people with access barriers to DAA treatment have been examined in our context, there are no known works that have studied to further understand this issue from the perspective of PWID. Therefore, this qualitative study aims to explore the access barriers for HCV detection and treatment in community health services from the perspective of PWID who are currently in a situation of imprisonment.

However, although the characteristics of people with access barriers to DAA treatment have been examined in our context, no work has been done to further understand this issue from the perspective of PWID sub-population entering prison.

## Methods

A qualitative study was carried out through semi-structured interviews [23] with PWID in prison at the Brians 1 Prison Centre (Sant Esteve Sesrovires, Barcelona). This is the pre-trial detention mixed prison in the province of Barcelona, with capacity for 1,600 people (1,300 men, 300 women). Pre-trial detention prisons are centres for the detention and custody of detainees and prisoners who have been remanded in custody during the processing of the court case, and serving sentences when the duration not exceed six months. In the prison where the study was carried out, 80% of the people admitted to prison come from the community, through which there is a high circulation of people between the prison and the community.

Barriers to accessing or not completing HCV diagnosis or treatment with DAAs in community health services were explored.

### Participants

In-person interviews were conducted with a sample of 33 people in prison who were active PWID. An intentional sampling was carried out with the objective of obtaining a sample that considered the participants’ variability according to socio-demographic characteristics and the phase in relation to HCV detection and treatment. Inclusion criteria included people who had not been detected to have HCV infection, who had not completed diagnosis or DAA treatment in the community before entering prison, as well as having been at freedom in the community in the last 6 months. Recurrent offenders were also recruited, among whom it was detected that having been released during the DAA treatment process, they had abandoned it during the last release period or had not carried out the opportune follow-up to confirm cure. People who presented deficient cognitive abilities or a strong language barrier that prevented them from carrying out the designed interview were excluded. The sample size was decided according to the saturation principle [24]; the team assessed that after having interviewed 33 people, no more new information was being obtained and stopped collecting data.

### Data collection

Data collection was carried out through a semi-structured interview [23] between November 2021 and April 2022. Initially, a first version of the interview was designed, which was initially tested with four participants. The questions were adapted after holding a meeting with all the members of the research team. The script of questions included topics related to the barriers to carrying out tests, for monitoring diagnosis and continuity in the hepatitis C treatment in community services at different levels: personal, regarding providers (health and non-health personnel) and in relation to the health system [25]. The interviews were carried out by four members of the team in interview rooms in the different prison residential wings, which allowed uninterrupted dialogue. They lasted between 30 and 45 min and were recorded digitally, in addition to taking notes throughout the interview. Socio-demographic data, penal situation, drug use and their current status in relation to HCV infection were also collected from the interviewees.

### Data analysis

The interviews were transcribed and analysed using the grounded theory approach, a form of qualitative research that operates inductively, allowing themes to emerge from the data [26, 27]. The transcriptions were assisted using NVivo 12 qualitative software to code them. In a first phase, the first and last author conducted a constant comparison analysis of the transcripts using open coding. The result of the coding was discussed in meetings with the whole research team in order to obtain more precise codes. This process was carried out periodically after the interviews were held and transcribed, and discrepancies and inconsistencies were detected, which facilitated more accurate coding. In the next phase, the transcripts were reviewed again, obtaining new codes, and previously obtained codes were merged or reformulated in order to make the coding more focused. Finally, themes were discussed and refined, leading to a list of codes arranged in categories and subcategories [27]. For quotations from the interview transcripts that illustrate the explanation of the themes obtained, the participant number, gender and nationality are indicated.

The credibility and validity of the data analysed were enhanced by triangulation at different levels [28]. In this study, data were collected from a heterogeneous sample of people, including men and women, nationals and foreigners, and of different ages, capturing different views among the population under study. These data were discussed and pooled by a team of researchers with different perspectives. The team was composed of 4 nurses with long experience in the prison environment (average of 11.5 years working in prisons), one of whom had a PhD in anthropology, and a medical doctor with expertise in public health.

### Ethics

All participants were informed of the research objectives, the confidentiality and anonymity criteria and the right to withdraw from the study. They were also informed that they would not receive any financial incentive for their participation. They signed an informed consent following Organic Law 3/2018, 5 December, on personal data protection and guarantee of digital rights and EU Regulation 2016/679. This project was approved by the Research Ethics Committee (REC) of the Institut Universitari d’Investigació en Atenció Primària Jordi Gol (IDIAP Jordi Gol), with CEIm code 21/168-P.

## Results

Thirty three participants were interviewed during the study, of whom six were women, one of them transgender. The average age of the sample was 38.6 years. Eighteen were of Spanish origin and 15 foreigners, 10 of whom were from Eastern European countries. 54.6% (18 people) had completed secondary education. All reported being in active use during their release stay, of whom 23 reported heroin and cocaine use (“speedball”) intravenously. Regarding harm reduction programmes, 19 were undergoing opioid substitution treatment (methadone) and 24 had attended harm reduction centres (HRC) to make use of DCR and NEP in the last six months. 57.6% (19 people) were diagnosed with HCV upon entering prison. Of those interviewees who started the DAA, six abandoned it before completation. Six finished treatments, but did not attend their follow-up appointment to confirm the SVR.

The relevant topics to determine the access barriers to diagnosing and treating HCV with DAAs were related to individual, social, community and political factors. Different concepts and categories were identified overall, among which there was an interrelation, which enabled a substantive theory to be generated. The results are presented on three levels: (a) personal, (b) health providers and (c) health system; that provide key information to understanding the access barriers to HCV diagnosis and treatment, useful information to guide the design of strategies that facilitate access for PWID sub-population entering prison.

### Barriers on a personal level

Participants described different barriers on a personal level, which fell into three categories: heavy drug use, lack of interest and knowledge about HCV and social exclusion.

### Heavy drug use

All the participants indicated that the main reason for not being able to comply with the HCV diagnosis and treatment process was that they were submerged in intense drug use. In general, the interviewees relate the use of intravenous drugs as a need that hinders a stability to decide to initiate or carry out HCV diagnosis and treatment.

*I haven’t gone anywhere while outside because the drug hasn’t left me. I knew where I could go, but the drug doesn’t allow me to do anything, not even call my family.* (Participant 28, male, Romania)

*You have the information, but we’re not here to undergo the treatment, we’re not responsible at that time. We just want the drugs. Not for treatment or anything.* (Participant 23, male, Spain)

Many of the PWID interviewed said that drug dependence was a condition that inhibits going for HCV treatment. In this sense, they expressed spending time immersed in consumer environments, looking for money to buy drugs to mitigate daily suffering.

*You’re not thinking of hepatitis, what you want is to get money and score drugs (buy drugs) and take them (consume). I was more interested in a hit (injecting) and “finding where the next one would come from” (find a way to make money).* (Participant 1, male, Spain).

*If you are consuming “smack” (heroin), all you think about is getting rid of the “cold turkey” (withdrawal syndrome), getting money, stealing.* (Participant 18, transsexual woman, Spain).

### Lack of interest and knowledge about HCV

Most of the participants had a lack of knowledge about HCV infection. In general, they had partial knowledge about the infection routes and preventive methods for HCV, the means to avoid blood-borne infections as no needle sharing or other injection material (spoons, filters, etc.). This fact was cited as a reason for not being interested in the serological status of infection or reinfection.

*I didn’t know how you could get hepatitis, HIV yes. I didn’t know anything about the treatment or how you get infected.* (Participant 22, male, Pakistan)

*In the one month I had been in Spain, I consumed and got infected. I think I got infected with the same drug or with the same syringe, I have never shared.* (Participant 3, male, Italy)

In this sense, in relation to the HCV symptoms, a large number of the participants expressed that they did not feel any symptoms that signified a serious health problem or that evidenced being infected, leading them to be interested in seeking treatment.

*It didn’t affect me when I was diagnosed. I stayed the same, normal. I was fine. I didn’t ask for the treatment.* (Participant 15, male, Georgia)

*I would have been worried if I had felt very screwed up in my body, that I felt in poor health. As long as you don’t feel anything, you don’t worry.* (Participant 1, male, Spain)

However, many of the participants were aware of being infected with HCV, but revealed other reasons for not starting treatment with DAAs. Close to one quarter reported not knowing about DAA treatment and the benefits of this treatment, as well as only having information on treatment regimens prior to this therapy (interferon and ribavirin).

*I knew nothing about the treatment, zero. They had told me about doing a biopsy, but I thought it wouldn’t heal and that one day or another I would die.* (Participant 27, male, Spain)

*I knew of a friend who had undergone the treatment and nearly died, so I didn’t want anything to do with the interferon.* (Participant 26, male, Spain)

In other cases, the fact that treatment with DAAs was considered safe and effective led to not considering HCV as an urgent or serious health problem and to perpetuating the decision to follow up with a doctor in order to access treatment.

*The truth is that I didn’t mind having Hepatitis C because I knew that I would undergo treatment and I would lead a normal life.* (Participant 23, male, Spain)

*When they gave me the diagnosis, I got very nervous, but since I have friends who have taken the pill and are fine… well, I didn’t worry much either.* (Participant 24, Male, Georgia).

### Social exclusion

Participants described living conditions that hinder their interest in HCV diagnosis and treatment. Two thirds of the sample reported not having a home, not being able to access shelters or being in homes irregularly, a situation that they describe as incompatible with HCV monitoring.

*I was living with a friend in a squat. I asked for a shelter and they told me there was a waiting list… I ended up on the street, I wasn’t there for treatment.* (Participant 12, male, India)

*Sometimes I slept in a squat and had to go to my methadone centre every day to take drugs in treatment (for HCV). I needed a boarding house so that I wouldn’t be left on the street, but they couldn’t get one for me.* (Participant 5, male, Iran)

Added to this situation, most of the participants reported not being able to be interested in their situation with HCV related to having other interests that they considered more important, such as having to attend court summonses related to criminal cases.

*Very good, but I had other problems on my mind… court cases, problems with the family, etc.* (Participant 32, male, Georgia)

*I wanted to start treatment when I had completed the things I had to do. I had legal problems, no housing, no documents to go to the hospital… (Participant 30, male, Serbia)*.

Among the foreign population interviewed, more than half described being in an irregular administrative situation as a problem in accessing treatment, which included not having a health card and not having facilities to access certain health services, as well as being unaware of these and other PWID-specific resources or programmes.

*I haven’t been to any doctor and I don’t know where to go on the street, I don’t have a health card either.* (Participant 24, Male, Georgia).

*They had stolen all my documents and I had nothing. They couldn’t give me the treatment. I never went to the doctor because I didn’t have a health card.* (Participant 33, female, Poland)

Five of the six women interviewed described being immersed in power relationships with their male partners as a barrier.

*Some offer you a “chuta” (syringe) exchange so that you are like them and this especially happens a lot from men to women, to keep you tied, so you are stigmatised like them and it is easier for you to be their girlfriend later. They hook you to them. It also happens with HIV.* (Participant 6, female, Spain)

*I had been with the same person for two years… I didn’t know that he had hepatitis. He hit me. I was diagnosed with hepatitis on my first injection. Later he was tested and he had hepatitis. They tricked me* (Participant 33, female, Poland).

### Barriers at the health provider level

Participants highlighted three issues related to health providers: receiving little information, not accessing screening and treatment and having poor interactions with health personnel.

### Receiving little information

Most of the participants reported not having information or not receiving it from the personnel in the health centres they attend (mainly HRC) about HCV infection and its treatment.

*They don’t give you any information. They don’t tell you about what it is and where you have to go. I didn’t know where I had to go.* (Participant 26, male, Spain).

*The doctors in Georgia and Spain didn’t give me any information. They didn’t tell me anything about the treatment either, only in prison.* (Participant 9, male, Georgia).

This fact was accentuated to a greater extent among foreign participants, especially those who had a strong language barrier.

*I was going to inject myself in the (…) DCR and I talked to the nurses. Before I spoke (Spanish) badly… now normal.* (Participant 4, male, Georgia).

*I didn’t understand Spanish. I only spoke to people from my own country.* I had no information about the people in the centre (health personnel) (Participant 12, male, India).

### Not accessing screening and treatment

Many of the participants reported that, despite going to HRCs or other drug dependence health services, they had not been offered HCV screening (rapid tests or blood tests) or, if applicable, they had not been proposed for follow-up to start DAA treatment.

*They never offered me the test. I went to consume in the consumption room, but they didn’t ask me anything.* (Participant 3, male, Italy)

*I used to go to my health centre to do tests, but never to look at hepatitis (C). They never offered me either at the Care Center for Substance Use (CCSU) or at the health care centre.* (Participant 22, male, Pakistan)

### Having poor interactions with personnel

Some the interviewees perceived having poor interactions with health service personnel, having difficulties in accessing HCV screening and follow-up.

*I noticed that my liver hurt and they referred me to the hepatologist. The hepatologist asked me “where are you going to pay for the burial?” It’s painful to hear a hepatologist telling you that.* (Participant 13, male, Spain).

*Professionals should be more open people and get involved in helping. Don’t always repeat the amount of money that everything costs. Sometimes they talk to you in bad ways.* (Participant 6, female, Spain).

### System level barriers

There were three issues related to the health system: complicated circuit, little comprehensive care, lack of community support.

### Complicated circuit

Most of the participants perceive different complications in accessing hospital services, where HCV diagnosis and follow-up is carried out through the usual circuit. Many of the participants indicated that the hospital services were so far away from their home or from the health services they regularly used (HRC or CCSU) that they did not attend the scheduled appointments, especially when they were early in the morning or delayed several days.

*From my CCSU, they sent me to the hospital to do tests, but it is a long way away from where I am. I never got to go. In the end I went to prison.* (Participant 25, male, Romania).

*They did tests on me at the hospital and they told me that I had to undergo treatment. I had to go to my CCSU every day and I lived a long way away in a squat. Many days I didn’t go.* (Participant 5, male, Iran)

In this sense, they also referred to the appointment times, perceiving appointment times scheduled at early hours or where there was a long time between follow-up visits as limiting.

*They offered me an appointment to take the test at 7 in the morning. I think that to do the analysis, if it had been at a time like 12, or 11, I don’t know, that would be fine for me, no problem.* (Participant 14, male, Spain).

*At the hospital they did a blood test and it took 45 days to give me the results. I went to prison for a few days and when I got out, I didn’t know where I had to go.* (Participant 15, male, Georgia).

Many of the participants expressed not knowing the health system, pointing out the complications to enter the circuit for HCV diagnosis and follow-up, especially among foreign people who don’t know the health system and people who were leaving prison.

*There are many documents and many appointments. I don’t know how appointments work and where I have to go to the hospital.* (Participant 28, male, Romania).

*When you go out on the street, there are many things and you find yourself very lost. I have often gone out with many documents that I don’t understand. There are many documents and it doesn’t surprise me that you get lost.* (Participant 1, male, Spain).

### Little comprehensive care

Many of the participants who go to drug dependence health services (HRC and CCSU) perceive that these services are focused on providing harm reduction programmes or treatment for drug dependence and don’t have HCV care.

*They should offer to do the tests in the consumption rooms (DCR). There they only give you syringes; they offer you methadone, but nothing to diagnose or treat you.* (Participant 21, male, Spain).

*You go to the consumption room (DCR) and they only give you syringes. You can shower and have something to eat, but they don’t pay attention to your real problems.* (Participant 8, male, Spain).

In this sense, many of the participants who were undergoing treatment for problems related to dependence highlighted not having the different treatments for problems concomitant to drug use unified in a single service as a limitation.

*What is needed is a good centre. We have consumption problems, health problems and we have nowhere to sleep. A good rehabilitation centre is needed that offers all kinds of help.* (Participant 23, male, Spain).

*I go to the CCSU for my treatment, but if you need social assistance or get tested for hepatitis or whatever, you have to go to other places. There is a lack of a place where they give you social assistance, drug treatment and they test you for everything.* (Participant 31, female, Spain).

### Lack of community support

Many of the participants mentioned not having sufficient support at the community level to undergo HCV monitoring and treatment. In this sense, they perceived that the stigmatisation from their families and relatives led to social isolation and not thinking about seeking treatment for HCV.

*You need support and love. When you’re lost, the family gets tired and despises you, that makes you want to get high and forget everything.* (Participant 20, male, Spain).

*They told me I had hepatitis. I went into depression. I received the news crying. I separated from my family because I didn’t want to infect them. My sister stopped talking to me. I don’t have any support.* (Participant 25, male, Romania).

The participants felt that there was insufficient campaigning to successfully involve people with HCV, perceiving that this health problem was not of interest to health institutions.

*I believe that for people to go for tests, they must be told everything they are going to lose if they don’t get treatment. You have to tell them politely, explaining it in detail and more clearly* (Participant 14, male. Spain).

*They need to campaign more, to involve people more in how serious hepatitis C is. I have seen many people die. More information is needed, more posters, more of everything… that gets through to you so that you want to undergo treatment.* (Participant 11, female, Spain).

## Discussion

Until now, no previous studies were known to have explored the barriers to PWID access to HCV diagnosis and treatment of DAAs in health services in community settings in Catalonia in depth. Previous quantitative studies indicated being young, a migrant, or having been released from prison as factors that hindered access to HCV diagnosis and treatment monitoring among PWID [8, 10, 11]. Based on this study, the insights of PWID sub-population entering prison have been analysed to better understand the perceived barriers, which broaden the knowledge of the barriers at different levels, to suggest a series of proposals to facilitate access to diagnosis and treating the HCV with DAA. This qualitative analysis revealed barriers in the personal, provider and health system level dimensions, many of which have been identified in recent reviews that included studies carried out with vulnerable populations similar to this study [5, 6]. However, this study has allowed us to incorporate the voice of the people studied in order to guide social and health interventions that are more in line with their needs and priorities [29, 30].

It was detected that PWID sub-population entering prison make use of drug use harm reduction programmes in specialised centres for this purpose, which offer and facilitate screening, HCV diagnosis and access to DAA treatment, although most of those studied were unaware of these services or did not enrol in them. The participants highlighted the fact of being immersed in intense drug use as the main perceived barrier to treatment. This aspect has been compiled in various qualitative studies that highlight drug use as an activity that weakens the stability to carry out treatment with DAA [6, 31, 32].

In relation to HCV, many of the participants were unaware of the transmission routes, the symptoms and treatment with DAAs and this turned out to be the reason for not being interested in the status of the infection and its treatment, a finding that has already been reported in other studies [32–37]. Many of the participants were aware of their serological status and the effectiveness of DAA treatments, although as in other studies, they stated that did not seek treatment for HCV because they did not consider it an urgent or serious problem [33, 35].

The participants in this study also identified a series of precarious social conditions that entailed multiple daily responsibilities that were a barrier to accessing HCV diagnosis and treatment that have been reported in other studies, such as the lack of regular housing [5, 38], social deficits and having problems with the law [6, 31, 36, 38], or in the case of women, being submerged in power relations with their male partners [39, 40]. In this study, it was detected that, in the migrant population, having an irregular administrative situation was a condition that represented a barrier to accessing health coverage, knowing the system and therefore limiting HCV diagnosis and treatment. The lack of knowledge of foreign PWID sub-population entering prison in relation to the possibility of health coverage is also denoted, through the exceptional circuit of the General Sub-directorate for Drug Dependence for cases that don’t have a health card [14].

Regarding the barriers with health providers, the participants reported having few interactions with the health personnel of the drug users’ services they attend. In this sense, as in other studies, the participants reported receiving limited information about HCV treatment and, consequently, they had no options to receive proposals for diagnosis with rapid tests or referrals to specialised services [5, 6, 41]. A significant number of PWID sub-population entering prison highlighted that they did not feel accepted by the personnel and reported receiving evasiveness and little advice about harm reduction services [6, 31].

In the case of foreign PWID with a strong language barrier, this situation was aggravated. This subpopulation reported not receiving information related to HCV prevention, diagnosis and treatment. If they did receive it, they reported difficulties in understanding the monitoring until starting treatment with DAAs. As has already been observed in other previous studies carried out in harm reduction services in Catalonia, a high percentage of foreign users is detected (around 40%) [9, 11], including people with strong cultural and language barriers, to receive information and health education related to the prevention of drug use and its consequences [19, 42].

The participants in this study expressed a series of inconveniences and limitations related to access and care from the health system as well as social support for HCV treatment and monitoring. In relation to specialised hepatology services, they referred to the inconvenience of scheduling appointments at early hours and the length of time between appointments, aspects that have already been reported in other studies [5, 43]. Allusion was also made to the difficulties in travelling to services far from their residence or the services they usually use (harm reduction services), an aspect detected in other studies [31–33].

In general, the participants expressed having other problems, health and social, which led to them having to go to multiple specialised care services. The PWID users of harm reduction services criticised that these services did not provide comprehensive care to their multiple social and health problems, including specialised HCV monitoring [5, 32, 36]. Participants who had abandoned HCV treatment after their release from prison expressed difficulties in addressing numerous challenges, such as going to appointments with social and health services [44], which made it difficult to carry out follow-ups and, consequently, to abandon treatment upon leaving prison [8]. A lack of community support was also expressed, by family members, relatives and peers, as well as the lack of campaigns that would promote greater involvement of society in the prevention and awareness of HCV treatment with DAA [6, 34, 36].

It was detected that the access barriers to HCV treatment with DAAs occur in subpopulations of PWID entering prison with intense drug use, with multiple structural problems and HCV infection knowledge deficits that reveal great challenges in interactions with health personnel and limitations to access the health system and receive social support. The findings of this study support the need to promote strategies with interventions at different levels to facilitate access to HCV diagnosis, treatment and monitoring. In order to respond to the specific needs of this group, comprehensive intervention is needed that articulates harm reduction programmes and treatment for drug dependence, multidisciplinary and specialised care for blood-borne infections and coverage of social needs, such as housing, access to health or care for legal needs [5, 6, 29].

The participants presented barriers to HCV follow-up and adherence to DAA related to heavy drug use. Despite efforts in our context to address this need through opioid substitution programs and treatments to manage stimulant drugs (cocaine, crack, etc.), our findings reveal the need to incorporate more flexible formulas tailored to the practices of PWID, based on the analysis of PWID entering prison. In this regard, it is recommended that heroin-assisted treatments, which have been shown to be effective in reducing the negative consequences of opioid use, increasing quality of life and reducing criminal activity be promoted [45, 46], as well as considering the promotion of opioids and stimulant safe supply to ensure a secure supply of substance that can improve health outcomes [47, 48]. These methods may favour the stabilisation of drug use and thus improve HCV follow-up and treatment with DDA.

The participants were people who had been in close contact with Barcelona’s drug consumption rooms. Previous studies [16, 49] point to DCRs as the ideal facility for promoting screening with rapid or analytical tests and for follow-up with DAA in confirmed cases. In connection with this, Barcelona’s drug consumption rooms need to be improved to accommodate health professionals who can provide HCV treatment within the local community. These services must offer a flexible approach to meet the needs and adapt to the social circumstances of PWID, with the aim of maintaining adherence to the community health system and achieving maximum detection, cure, and monitoring of HCV. In line with this idea, there is a need to implement specialised training for professionals, in order to equip them with the skills needed to communicate and engage with PWID. Previous studies [5, 6, 36] point out that provider training improves HCV knowledge, increases confidence in treating PWID and leads to greater sensitivity when dealing with users’ structural and cultural specificities. In addition, the mobilisation of prevention campaigns with peer workers is recommended to facilitate access to DAA treatment. Previous studies [50, 51] suggest that greater investment in promoting peer education approaches to improve support pathways, reduce stigma and improve access to HCV and drug treatment among PWID is a priority. In this vein, peer support interventions have proven successful in overcoming social, cultural and linguistic barriers between staff and foreign patients [50, 51].

The participants in this study suffered from deficits in social circumstances related to a lack of regular housing, administrative irregularities affecting migrants, and power dynamics affecting women. PWID entering prison face barriers to HCV follow-up and DAA treatment due to a lack of stable housing. In this regard, the evidence supports the promotion of housing first with harm reduction approaches that could bolster treatment entry, engagement, retention and successful completion for both drug use and blood-borne infection follow-up [52].

Our findings show that migrants with irregular immigration statuses have difficulty accessing the health care system and HCV treatment coverage. In this context, support strategies that address social and public health needs more broadly must be promoted, and policies to address migrant vulnerability must be strengthened to encourage healthy coping mechanisms and harm reduction for intravenous drug users in at-risk settings [53].

Lastly, to improve access to HCV treatment with DAA, more attention needs to be paid to the criminalisation and stigmatisation of PWID. In this regard, as several studies [29, 46, 54] point out, better identification of the subgroups that present barriers to DAA treatment and drug use treatments is needed. In our case, we found that migrants, women and people with criminal records persistently present barriers related to police presence and social rejection at an individual, community or structural level. From this perspective, harm reduction services must be optimised to inspire trust and promote policies that strengthen social and family support and reduce criminalisation among these sub-populations [29, 54].

### Limitations

This study may be subject to a number of limitations related to the characteristics of the sample. The research was carried out in a unique prison in Catalonia, although this was the unique appropriate centre to recruit a sample of pre-trial detainees who had recently been in a situation of freedom, as the study aims to look for barriers in the community. The sample size is not large, but throughout the data collection and analysis data saturation was quickly reached and it was considered it unlikely that increasing the sample would affect the research findings. Users selected directly from community services did not participate. However, it is known that a good proportion of drug users have problems with the law. Therefore, people first-time admitted in prison were included in the sample who told us about their recent experiences in freedom, which provided key information. Also, the participation of women was low, given that there is a lower percentage of women who are PWID and who are in prison [9, 55]. People with severe mental health problems, who are outside of the domain of the research team, were not included. Despite these limitations, the findings of this research can provide important guidelines to facilitate access to diagnosis and treatment in community health services for PWID with hepatitis C. In-depth studies in the future in subpopulations that could not be accessed or had less participation are recommended.

## Conclusions

While strategies for HCV diagnosis and treatment with DAAs have been intensified and made more accessible in Catalonia, PWID continue to face barriers in accessing the health services available to them in the community. Knowing the barriers to accessing HCV treatment with DAA in the community environment based on the experiences of PWID sub-population in pre-trial detention prison offers key information to improve the design of future strategies. These experiences reveal some subpopulations of the PWID collective with strong barriers that suggest that the scope of HCV treatment from community health services could be intensified. In this study, personal barriers were detected that are related to intense drug use, lack of interest and knowledge of HCV infection and treatment and a situation of extreme social exclusion among PWID. These people encounter barriers with health providers related to receiving little information, the language barrier, not receiving proposals for HCV screening and treatment and having poor interactions with health personnel. They also expressed finding systemic barriers related to considering the circuit of the health system complicated, perceiving care that was not comprehensive and the lack of community support. In the future, efforts must be made to promote prevention and treatment and integrate harm reduction strategies into social policies to provide multilevel care for intravenous drug users who have been diagnosed with HCV. This will require advocating for drug substitution programmes that are more flexible than those currently employed, reinforcing diagnosis and treatment by bringing specialised health services closer to harm reduction centres, improving health education with more training for health personnel and the inclusion of peer education interventions, as well as providing more community support and social care, including access to housing and policies to reduce the stigmatisation and criminalisation of PWID.

## Data Availability

The datasets generated during the current study are not publicly available due to personal and biographical data and no English translated interviews but are available from the corresponding author on reasonable request. All data analysed during this study are included in this published article.
